# Redescription of *Paradiplozoon hemiculteri* (Monogenea, Diplozoidae) from the type host *Hemiculter leucisculus*, with neotype designation

**DOI:** 10.1051/parasite/2018004

**Published:** 2018-02-09

**Authors:** Dagmar Jirsová, Xuejuan Ding, Kristína Civáňová, Eliška Jirounková, Jana Ilgová, Božena Koubková, Martin Kašný, Milan Gelnar

**Affiliations:** 1 Department of Botany and Zoology, Faculty of Science, Masaryk University, Kotlářská 2, 611 37 Brno Czech Republic; 2 Department of Forest Botany, Dendrology and Geobiocoenology, Faculty of Forestry and Wood Technology, Mendel University in Brno, Zemědělská 3, 613 00 Brno Czech Republic; 3 School of Life Science, South China Normal University, Guangzhou, 510631 People’s Republic of China; 4 Department of Parasitology, Faculty of Science, Charles University, Viničná 7, 128 44 Prague Czech Republic

**Keywords:** Monogenea, China, fish parasite, neotype, species identification, phylogenetic analysis

## Abstract

*Paradiplozoon hemiculteri* (Ling, 1973), a member of the Diplozoidae, parasitizes the gills of Asian fish. Not only is the type material unavailable for this species, the original description was poor and somewhat conflicting, and adequate molecular data were not available. What is more, the available morphological and molecular data are inconsistent and fluctuate significantly. Here, we present a redescription of *P. hemiculteri* based on morphological and molecular data from new isolates collected from the type host, the sharpbelly *Hemiculter leucisculus* (Basilewsky, 1855), captured at the neotype locality (Shaoguan, Guangdong Province, southern China); a neotype for *P. hemiculteri* was designated from this collection. The length and width of the body, buccal suckers, pharynx, attachment clamps, sickle and the central hook handle were all measured and the shape of the anterior and posterior part of the median plate and anterior and posterior joining sclerites accurately documented. Phylogenetic analyses based on the sequences of the second rDNA internal transcribed spacer (ITS2) indicated that all new samples clustered together and differed clearly from sequences attributed to *P. hemiculteri,* which are deposited in GenBank. Our results confirm that *P. hemiculteri* is the only diplozoid that has demonstrably been found on the gills of *H. leucisculus* to date.

## Abstract (fr)

*Paradiplozoon hemiculteri* (Ling, 1973), membre des Diplozoidae, parasite les branchies des poissons asiatiques. Non seulement le matériel-type n’est pas disponible pour cette espèce, mais la description originale était médiocre et quelque peu contradictoire, et des données moléculaires adéquates n’étaient pas disponibles. Qui plus est, les données morphologiques et moléculaires disponibles sont incohérentes et fluctuent de manière significative. Ici, nous présentons une nouvelle description de *P. hemiculteri* basée sur des données morphologiques et moléculaires provenant de nouveaux isolats recueillis chez l’hôte-type, *Hemiculter leucisculus* (Basilewsky, 1855), capturé à la localité du néotype (Shaoguan, province du Guangdong, sud de la Chine); un néotype pour *P. hemiculteri* a été désigné à partir de cette collection. La longueur et la largeur du corps, les ventouses buccales, le pharynx, les pinces, la faucille et la poignée centrale ont été mesurés et la forme de la partie antérieure et postérieure de la plaque médiane et des sclérites antérieurs et postérieurs bien documentée. Des analyses phylogénétiques basées sur les séquences du second espaceur transcrit interne de l’ADNr (ITS2) ont indiqué que tous les nouveaux échantillons étaient regroupés et différaient clairement des séquences attribuées à *P. hemiculteri* qui sont déposées dans GenBank. Nos résultats confirment que *P. hemiculteri* est le seul Diplozoidae qui ait été trouvé sur les branchies de *H. leucisculus* à ce jour.

## Introduction

Members of the class Monogenoidea (parasites of salt and freshwater fish) can be classified into two subclasses: Polyonchoinea and Heteronchoinea [4]. Within the Heteronchoinea (infrasubclass Oligonchoinea), the Diplozoidae Tripathi, 1959 represents a specific monogenean group parasitising the gills of freshwater fishes. Diplozoids are obligatory blood-feeding ectoparasites with an unusual life cycle wherein two immature individuals (diporpa) meet on the gills of a fish and subsequently permanently fuse together into an X-shaped adult ’individual’ [18,35]. Each adult worm (i.e. each ’side’ of a fused individual) has a forebody and an ovarium and testis in each hindbody. The adult has two attachment apparatuses, each having four pairs of clamps and a pair of small central hooks situated on the ventral side of the respective opisthaptors.

According to Khotenovsky (1985) [18], the Diplozoidae is represented by two subfamilies: Diplozoinae Palombi, 1949 (five genera) and Neodiplozoinae Khotenovsky, 1980 (two genera). Morphological features (e.g. size of body, size of clamps) vary widely within diplozoid species, depending on size of the host fish and the developmental stage of the diplozoon [23,24], making determination to species level difficult. In general, the central hooks and clamp sclerites are considered the structures of most taxonomic relevance [5,11,18,22]. In more recent years, genetic analysis of molecular markers has been used to counter morphological similarities when differentiating species [21–23,34]. It is widely assumed that the majority of diplozoids are highly specific to their hosts; indeed, most follow their specific fish host throughout their distribution range and they are very likely to have co-evolved together [17,38]. As such, host-species determination has often been used for general identification of the parasite [16]. One exception to this general ’rule’ is the generalist parasite *Paradiplozoon homoion* (Bychowsky & Nagibina, 1959), which has been recorded from more than fifteen cyprinid fish species [10,18,21].

In China, 33 species have been reported from five diplozoine genera, 23 from *Paradiplozoon*, six from *Sindiplozoon,* two from *Inustiatus,* one from *Diplozoon*, and one from *Eudiplozoon* [3,9,36,39]. Several authors [9,39] have published information on Chinese diplozoid species, including the original description of *Paradiplozoon hemiculteri* by Ling (1973); however, the morphological descriptions in these studies have largely been imperfect and/or inaccurate e.g. Chen (1973) [6] or the studies were based on molecular data only [9]. Despite all the published work concerning Chinese diplozoons, the molecular data are not accurate and none of the published studies adequately combined and reviewed morphological and molecular data [22,7,1,31–33]. Moreover, according to the curator of the collection, Mr. Weijian Yao, and specialists from the Institute of Hydrobiology, Chinese Academy of Sciences, where the type material of *P. hemiculteri* should be located, the types of this species are missing and likely lost. Therefore, we collected new material from Shaoguan, Guangdong Province neotype locality and type host, from which a neotype for *P. hemiculteri* was selected and designated. In this work, we redescribe *P. hemiculteri* based on the new morphological and molecular data from the neotype and new specimens from the type host in southern China.

## Material and Methods

### Sampling

As the type specimens of *P. hemiculteri* are now unavailable, the present study is based on morphological and molecular examination of ten adult diplozoid worms collected from the gills of *Hemiculter leucisculus* (Basilewsky, 1855), the type host fish species, from the neotype locality in Shaoguan, Guangdong Province, southern China (24.810329°N 113.597547°E) in 2015. Two individuals already fused into the X-shaped adult worm were separated, one haptor being fixed in 96% ethanol for molecular investigation. The second haptor was placed on a slide in a drop of water, covered with the cover slide, while excess water was drawn off with filtration paper until rupture of the parasite’s body appeared, and the sample was infiltrated/fixed with ammonium picrate-glycerine 1:1 (GAP) [8,20] for morphometric analysis.

### Morphological analysis

After the fixation of the haptor in GAP, the sclerites of diplozoid clamps laid nearly in one plane, enabling accurate measurement.

An Olympus BX51 light microscope (Olympus, Japan) equipped with differential interference contrast and Stream Motion digital image analysis software v. 1.5 was used to measure the length and width of the body, buccal suckers, pharynx, attachment clamps and the central hook sickle and handle [18]. All measurements were performed 8-16 times. An Olympus U-DA drawing attachment was used to create outline drawings of the central hook clamps and sickle. The haptoral terminology used herein follows Pečínková et al. (2007) [26].

### Molecular analysis

The DNA from ten samples was isolated according to the protocol published by Zavodna et al., (2008) [40]. Sequencing of the second rDNA internal transcribed spacer (ITS2) was used for molecular identification, using PCR primers adopted from Bachellerie & Qu (1993) [2]. The PCR reaction (50 μl) for amplification of the ITS2 region consisted of 5 μl 2x High Fidelity PCR Buffer (Thermo Scientific), 5 μl 25 mM MgCl_2_, 2 μl 10 mM dNTP, 1 μl (5 U/μl) of Taq DNA polymerase (Thermo Scientific), 32 μl PCR H_2_O, 2 μl 10 μM specific forward primer (DITS2F 5́-GGCTYRYGGNGTCGATGAAGAACGCAG-3́) and reverse primer (DITS2R 5́-GCCGGATCCGAATCCTGGTTAGTTTC TTTTCCT-3́), and 1 μl (50 ng) of DNA template. Amplification took place in a MyCycler thermal cycler system (Bio-Rad, USA) using the following protocol: 94 °C for 2 min; 30 times 94 °C for 30 s, 58 °C for 30 s, 72 °C for 60 s and a final 10 min extension at 72 °C. The PCR products obtained were separated on agarose gel (1%), stained with GoldView (Dongsheng Biotech), purified using a High Pure PCR Product Purification Kit (Roche) and cloned onto *Escherichia* *coli* TOP10 using the CloneJET PCR Cloning Kit (Thermo Scientific), according to the manufacturer’s protocol. Colonies were subsequently checked for fragment presence using PCR, with positive plasmids isolated using the High Pure Plasmid Isolation Kit (Roche) and sequenced in both directions using DITS2F and DITS2R PCR primers. The individual sequences obtained were compared with the NCBI database using the Basic Local Alignment Search Tool (BLAST).

### Phylogenetic analysis

All sequences were aligned and manually edited using Geneious software v 6.1.8 [17]. The final dataset consisted of our data and all available *Paradiplozoon* spp. sequences from GenBank (Table 1), the whole dataset being aligned using the MAFFT software package [15]. The most suitable nucleotide substitution model was chosen according to Akaike Information Criteria using jModeltest software v 2.1.4 [27]. Phylogenetic analysis was performed on the model with the best likelihood score (GTR + G) on MrBayes v 3.2.2 Bayesian inference software [13,30], using 20 million Markov chain Monte Carlo generations with four chains and four independent runs. Convergence of runs was checked using AWTY software (Are We There Yet [25]) in order to estimate burn-in. Maximum-likelihood phylogeny was performed under the same model using PHYML software v 3.0 [12]. The reliability of branching patterns within trees was tested by the bootstrap method with 1,000 re-samplings. The final trees were visualised in FigTree software v 1.4.2 [28]. The sequence of closely related *Inustiatus inustiatus* (DQ098893) was used as the outgroup for both phylogenetic analyses (see Table 1).

**Table 1:** GenBank ITS2 sequences of monogenean species selected for phylogenetic analysis. *Paradiplozoon hemiculteri* GenBank sequences are marked with an asterisk; data obtained for this study are also underlined. Outgroup sequence of *Inustiatus inustiatus* is in bold.

NCBI number	Species
AF369759	*Diplozoon paradoxum*
AF369760	*Diplozoon homoion*
AF369761	*Diplozoon bliccae*
AJ300710	*Eudiplozoon nipponicum*
AJ300711	*Paradiplozoon megan*
AJ300712	*Paradiplozoon bliccae*
AJ300713	*Paradiplozoon sapae*
AJ300714	*Paradiplozoon pavlovskii*
AJ300715	*Paradiplozoon homoion*
AJ563371	*Paradiplozoon nagibinae*
AJ563372	*Diplozoon paradoxum*
DQ098882	*Paradiplozoon parapeleci*
DQ098883	*Paradiplozoon parabramisi*
DQ098884	Paradiplozoon hemiculteri*
DQ098885	*Paradiplozoon jiangxiensis*
DQ098886	*Paradiplozoon parabramisi*
DQ098887	Paradiplozoon hemiculteri*
DQ098888	Paradiplozoon hemiculteri*
DQ098889	*Paradiplozoon parabramisi*
DQ098890	*Paradiplozoon opsariichthydis*
DQ098891	*Paradiplozoon diplophyllorchidis*
DQ098892	Paradiplozoon hemiculteri*
DQ098898	*Sindiplozoon ctenopharyngodoni*
HE653910	*Paradiplozoon bingolensis*
HF566124	*Paradiplozoon ichthyoxanthon*
HG423142	*Paradiplozoon vaalense*
KP340972	*Paradiplozoon homoion*
KP340973	*Paradiplozoon gracile*
KP340974	*Paradiplozoon skrjabini*
KY124645	Paradiplozoon hemiculteri*
KY124646	Paradiplozoon hemiculteri*
KY124647	Paradiplozoon hemiculteri*
KY124648	Paradiplozoon hemiculteri*
KY124649	Paradiplozoon hemiculteri*
KY124650	Paradiplozoon hemiculteri*
KY124651	Paradiplozoon hemiculteri*
KY124652	Paradiplozoon hemiculteri*
KY124653	Paradiplozoon hemiculteri*
KY124654	Paradiplozoon hemiculteri*
KY290757	Paradiplozoon hemiculteri*
KY290758	Paradiplozoon hemiculteri*
KY290759	Paradiplozoon hemiculteri*
KY290760	Paradiplozoon hemiculteri*
KY290761	Paradiplozoon hemiculteri*
LC050522	*Paradiplozoon skrjabini*
LC050523	*Paradiplozoon skrjabini*
LC050524	*Paradiplozoon skrjabini*
LC050525	*Paradiplozoon skrjabini*
LC050526	*Paradiplozoon skrjabini*
LC050527	*Paradiplozoon skrjabini*
LC050529	*Paradiplozoon skrjabini*
LT574865	*Paradiplozoon krugerense*
**DQ098893**	***Inustiatus inustiatus***

## *Paradiplozoon hemiculteri* (Ling, 1973)

Type host: *Hemiculter leucisculus* (Basilewsky, 1855)

Site: Gills

Original type locality: Huanggang, Hubei Province, People’s Republic of China (30.44°N, 114.87°E).

Original type material: The holotype originally described by Ling, 1973 is now unavailable (catalogue number unavailable).

Neotype locality: Shaoguan, Guangdong Province, People’s Republic of China (24.810329°N, 113.597547°E).

Type material: Type specimens apparently lost. Official collection where the types of *P. hemiculteri* should be located: Laboratory of Fish Parasitology, Institute of Hydrobiology, Chinese Academy of Sciences, People’s Republic of China.

Neotype material: The IPCAS M-565 *P. hemiculteri* neotype and three voucher specimens are deposited at the Institute of Parasitology, Czech Academy of Sciences, České Budějovice, Czech Republic. Three further voucher specimens are deposited at the Laboratory of Fish Parasitology, School of Life Science, South China Normal University, People’s Republic of China.

Article 75 of International Commission on Zoological Nomenclature was thoroughly followed.

### Redescription (Figs. 1a,b; 2a,b) 

**Figure 1:**
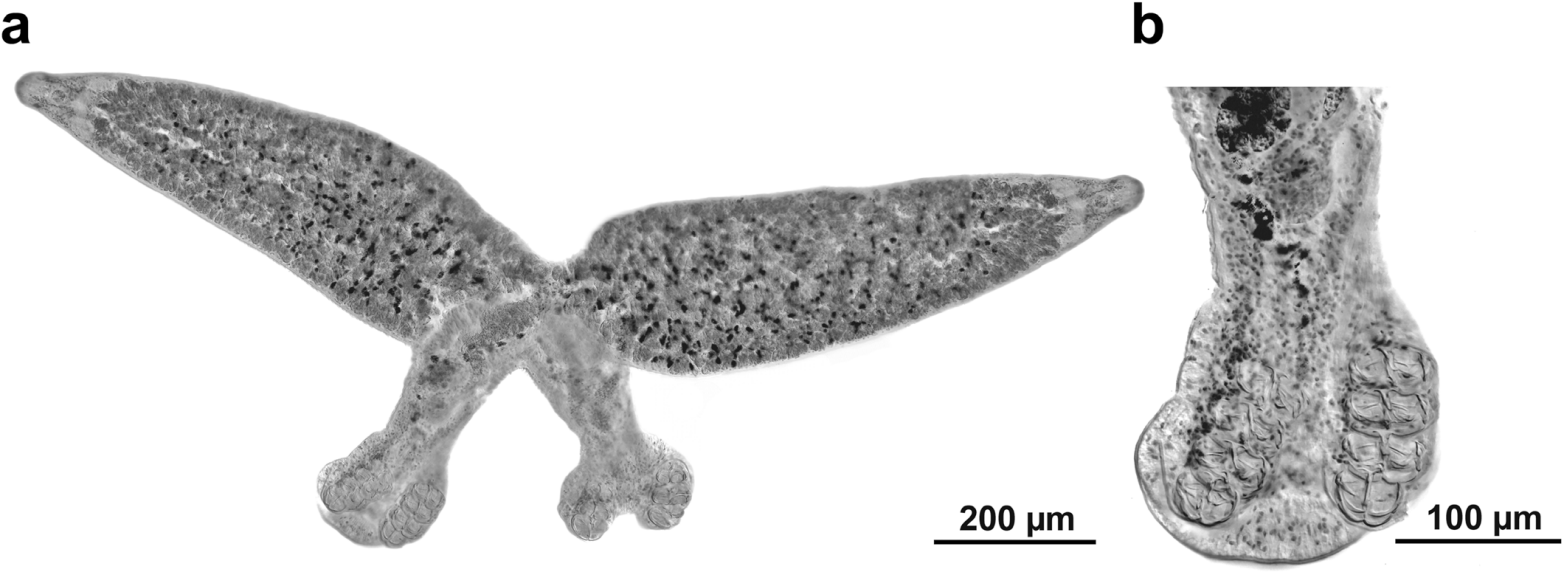
*Paradiplozoon hemiculteri*. A) Overall total; B) Detail of the two rows of clamps on the opisthaptor.

Adult Diplozoidae (Diplozoinae with *Paradiplozoon* characteristics [18]) display a typical X-shaped body, divided into a fore- and hindbody (Fig. 1a).

**Figure 2:**
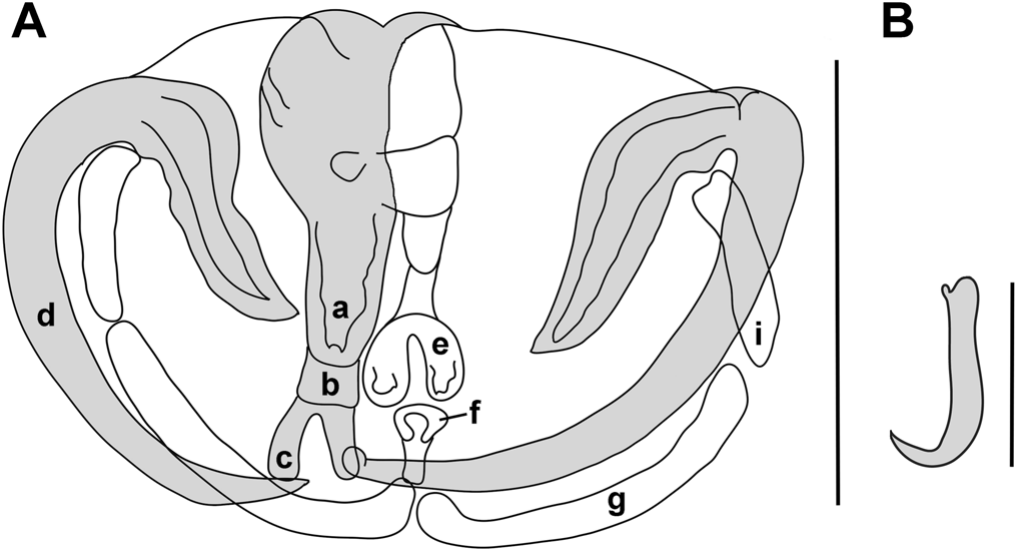
*Paradiplozoon hemiculteri* clamp. A) Clamp morphology (scale bar = 50 μm); a) anterior end of median plate, b) trapeze spur, c) anterior joining sclerite, d) proximal tip of anterior jaw, e) posterior end of median with a wide rounded sclerite, f) posterior joining sclerite, g) medial sclerite of posterior jaw, i) lateral sclerite of posterior jaw. B) Central hook sickle (scale bar = 20 μm).

Total body length, including haptor, 3153 μm (range 2320-4180, n = 8); maximum body width 931 μm (729-1140, n = 8). Oral opening sub-terminal, pair of oval-shaped buccal suckers of 58 (50-67) x 54 (42-65) μm (n = 16), located ventrally at the anterior part of the body, near to the opening of a muscular pharynx; pharynx 66 (50-83) x 54 (38-62) μm (n = 16). Branched intestinal caeca located in the forebody, though some branches reach the hindbody near the attachment apparatus. Intestinal caeca run in all directions. Vitellaria present in the forebody; ovarium and testis located in the hindbody along with a clearly visible ootype. Eggs were not observed.

The opisthaptors in the adult worm each comprise four pairs of clamps organised into two rows with two central hooks between them (Fig. 1b, 2a). The first (anterior-most) and smallest clamp is 56 μm (42-70) long and 90 μm (72-119) wide (n = 16), the second 60 μm (49-69) x 104 μm (91-115) (n = 16), the third 61 μm (47-82) x 107 μm (74-127) (n = 16), and the fourth 62 μm (47-82) x 105 μm (76-126) (n = 16).

The clamps are formed by fine sclerites. The anterior end of the median plate is rectangular with rounded corners and a narrow trapeze spur connected to the anterior end of the plate. The anterior joining sclerite, connected to the proximal tip of the anterior jaw, has the typical inverted V-shape. The posterior end of the median plate narrows and terminates with a wide rounded sclerite with an opening. The posterior joining sclerite is the same length as the anterior joining sclerite.

The central hooks are situated near the first (anterior-most) pair of clamps. The length of the central hook sickle was 17.6 μm (17.0-18.1) (n = 16) and handle length was 36.6 μm (34-37.9) (n = 16). A barely visible wing covers the blade. Complete drawings of the third clamp and central hook of the neotype are provided in Figs. 2a, 2b.

### Molecular analysis

DNA sequences isolated and amplified from the ITS2 fragment (762 bp) of ten adult worms were all similar, with less than 1% variability. The ten sequences have now been deposited in GenBank under accession numbers KY124645 − KY124654.

### Phylogenetic analysis

**Figure 3:**
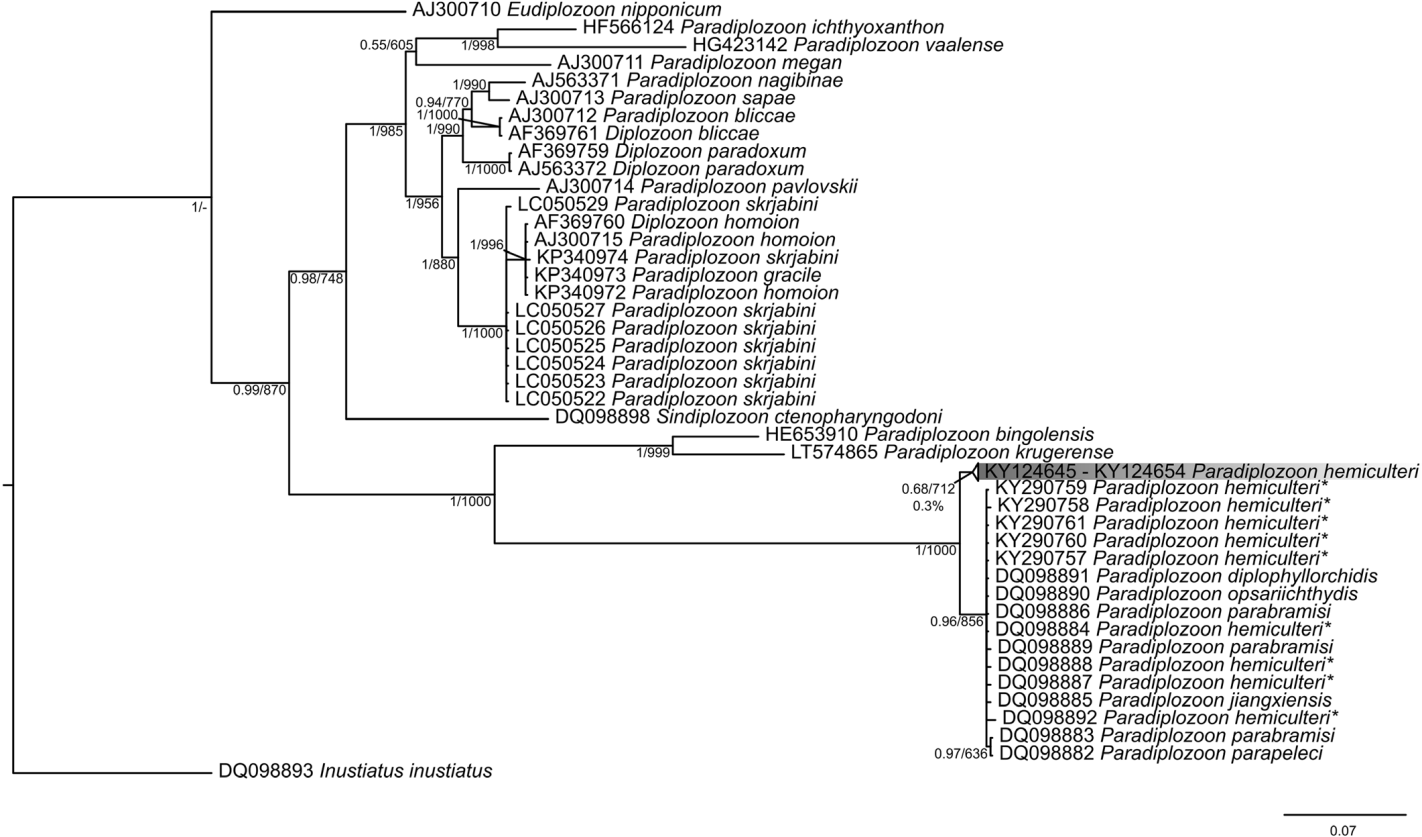
Concatenated phylogenetic tree based on ITS2 sequences for selected diplozoids. Constructed using MrBayes software, the tree includes results for Bayesian inference and maximum-likelihood with PP/bootstrap branch support. Genetic distance between our sequence data is listed below the branch support. Sequences of *Paradiplozoon hemiculteri* previously stored in GenBank are marked with an asterisk. Data obtained for this study are highlighted by grey gradient.

As the results of Bayesian inference and maximum likelihood analysis were almost identical, we pooled the results into one figure. The final tree is based on Bayesian inference phylogenetic tree topology with branch supports stated for both analysis types (bootstrap and posterior probability; Fig. 3).

All our samples clustered together and created a well-supported and separated group from those of *P. hemiculteri* sequences deposited in GenBank. Genetic distances between our sequences ranged between 0.1 and 0.3% (Fig. 3). Interestingly, all *P. hemiculteri* sequences deposited in GenBank (DQ098884 [host: *Hemiculter leucisculus*], DQ098886 [host: *H. leucisculus*], DQ098887 [host: *H. leucisculus*], DQ098892 [host: *H. leucisculus*], KY290757-61 [host: *H. leucisculus*]) clustered with different *Paradiplozoon* species (*P. diplophyllorchidis*, *P. opsariichthydis*, *P. parabramisi*, *P. jiangxiensis* and *P. parapeleci*), respectively.

## Discussion

In reviewing the available literature and corresponding sequence data deposited in public databases, we observed that taxonomic classification of the Asian diplozoon *P. hemiculteri*, a parasite of the gills of *H. leucisculus*, remains somewhat controversial at this time. Morphological descriptions of diplozoons isolated from *H. leucisculus* tend to be either inaccurate or inconsistent, with published illustrations of the clamps and central hook often ignoring key characteristic markers [9,18,39]. Furthermore, some of the sequences available in GenBank (i.e. DQ098884, DQ098887, DQ098888, DQ098892, KY290757-61) are probably misnamed as *P. hemiculteri*. In order to address this, we performed parallel morphological and molecular analyses on ten samples collected from the gills of the original type host *H. leucisculus* caught at the neotype locality, Shaoguan, Guangdong Province, southern China.

Based on our re-evaluation of the major morphological features (length and width of the body, buccal suckers, pharynx, attachment clamps, sickle and central hook handle), we determined that our specimens represent *P. hemiculteri* naturally parasitising the type host *H. leucisculus*. We evaluated our redescription based on published results of other authors [6,18,39]. While previous drawings of the main morphological characteristics (e.g. the two rows of clamps) were neither consistent nor always accurate, we were able to compare them to our own results and thereby distinguish our samples from other diplozoons. Unfortunately, the relevant type material of *P. hemiculteri* is now unavailable, hence further morphological comparison was impossible.

Due to the inconsistencies in previously published morphological data on *P. hemiculteri*, we decided to undertake additional molecular analysis based on amplification of the 762 bp ITS2 fragment from DNA templates of ten adult worms (see above). Our sequence data did not match any sequence saved in GenBank, including some sequences already assigned as *P*. *hemiculteri* (GenBank accession numbers DQ098884, DQ098886, DQ098887, DQ098892, KY290757-61 [9]). The genetic distance between ITS2 sequences in our dataset was almost negligible, ranging from 0.1-0.3% (Fig. 3), supporting the assumption that all sequences are related to the same diplozoid species. Recently, Gao (2007) [9] also performed a study on diplozoons originating from *H. leucisculus*; however, they used molecular methods and sequence data only, without supplementary morphological analysis. The absence of such morphological parameters could have led to incorrect assignment to species. Furthermore, phylogenetic analysis of the majority of *P. hemiculteri* nucleotide sequences available in public databases (before our own input; marked with an asterisk in Table 1, Fig. 3) indicates that all sequences clustered into one clade together with other species. Moreover, according to the phylogenetic analyses, we might assume that *Paradiplozoon* is paraphyletic as was also shown in the work of Gao (2007) [9]. However, these results would need revision because the relevant evaluation of potential paraphyly in the *Paradiplozoon* group cannot be based on the single *Sindiplozoon ctenopharyngodoni* sequence included in the analysis.

Based on the high morphological similarity and degree of homology in their genomes, it is possible that *P. hemiculteri* displays high species complexity. Similar high species complexity has been shown in marine capsalid monogeneans [37] and freshwater gyrodactylid monogeneans [14]. Both these studies concluded species complexity based on detailed knowledge of the species’ morphological features, combined with high marker similarity during molecular analysis. However, our molecular data could also suggest the existence of cryptic species within *Paradiplozoon*, as has recently been discovered in other monogenean genera such as *Gyrodactylus* [19,29]. In these studies, while the main morphological characters of the *Gyrodactylus* species/lineages (anchor hooks, marginal hooks, ventral bars, marginal hook sickles) were difficult to separate, molecular data (complete *cytochrome oxidase I* and ITS2 genes) clearly indicated different genotypes of cryptic species. The lack of published morphological descriptions for Chinese diplozoons is a major hindrance for this study as it means these hypotheses cannot be reliably confirmed or refuted at this time. This highlights the importance of combining accurate morphological analysis with molecular analysis of markers as the main means of species identification.

In conclusion, this study provides an accurate redescription of *P. hemiculteri*, thereby addressing the absence of type material and inaccuracies in the original illustrations and sequence clasifications that have been the norm up until now. Our results confirm *P. hemiculteri* (Diplozoidae) as the only diplozoid demonstrably parasitising the gills of its Asian fish host, *H. leucisculus*.

### Conflict of interest

The authors declare that the research was conducted in the absence of any commercial or financial relationships that could be construed as a potential conflict of interest.
